# Swinging rhythms

**DOI:** 10.1007/s12471-022-01695-7

**Published:** 2022-05-10

**Authors:** J. Dijkmans, J.-T. Wijmenga, R. Tukkie

**Affiliations:** 1 Department of Cardiology, Amsterdam University Medical Centre, location VUmc, Amsterdam, The Netherlands; 2 Department of Cardiology, Spaarne Hospital, Haarlem, The Netherlands

## Answer

What stands out in the second electrocardiogram (Fig. 2) is the rhythm’s bidirectional pattern with a beat-to-beat shift in the QRS axis from −60 to +120 degrees and complete left bundle branch block with a QRS duration of 160 ms. A supraventricular origin with aberrant conduction is excluded as the rhythm is preceded by atrial fibrillation without tachycardia-dependent aberrancy when shorter R‑R intervals are present. Bigeminy is excluded by the regularity of the QRS intervals. The persistent left bundle branch morphology points to a ventricular tachycardia originating in the His-Purkinje system with alternating conduction from the left anterior to the left posterior fascicle. Bidirectional ventricular tachycardia (BVT) is the correct diagnosis.

In most cases of BVT, an aetiology that causes triggered activity is present [1]. In this case, there is no evidence for digoxin toxicity, electrolyte disturbances, myocarditis or other aetiologies that have previously been associated with BVT. However, an almost identical electrocardiogram has been published that was recorded in a decompensated 26-year-old woman with dilated cardiomyopathy in the absence of any other cause for triggered activity [2]. Further, acute heart failure is known to cause non-bidirectional ventricular tachycardia by triggered activity due to calcium overload as well, and an increased risk for digoxin toxicity has been reported in heart failure patients with a low digoxin plasma concentration (1.2–2.0 µg/l) [3]. Therefore, the most likely mechanism for BVT in this patient is intracellular calcium overload after administration of a digoxin loading dose during acute heart failure, causing delayed afterdepolarisation and triggered activity. Because of the severely depressed left ventricular ejection fraction no beta-blocker was administered. The arrhythmia terminated spontaneously.
